# Far-right music events, protests, and violence: a county-level dataset for Germany, 2013–2024

**DOI:** 10.1038/s41597-026-08252-6

**Published:** 2026-09-02

**Authors:** Markus Lang, Lotta Mayer

**Affiliations:** Max Weber Institute of Sociology, Heidelberg University, Heidelberg, Germany

## Abstract

Far-right activism materializes in music events, marches, and violence. Yet, the empirical links between these types of activism remain unclear. Quantifying these manifestations is difficult because of fragmented reporting and inconsistent geocoding at the local level. We introduce the Far-Right Activism (FARAC) dataset, a panel (2013–2024) that aggregates German federal parliamentary inquiries and civil society records at the county level. The dataset addresses the problem of regional assignment by standardizing all event records in accordance with 2024 administrative boundaries. This harmonized structure permits the first systematic, local-level comparison of far-right activism. It uses standard administrative identifiers, enabling direct integration with electoral results, sociodemographic indicators, and other county-level datasets.

## Background & Summary

The far right can best be understood as a social movement that also fields its own movement parties, and hence displays a very broad set of practices^1,2^. Quantitative research into far-right activities, however, has been both selective and fragmented, limiting our understanding of the patterns of far-right activism. Drawing on the work of Simi *et al*.^3^ on white-supremacist activism, while stressing the instrumental alongside the expressive character of these practices, we define far-right activism as the diverse set of practices (collective or individual, organized or not) that express or promote far-right ideology, emotions, and collective identity, or that serve to advance the far right’s goals. We focus on protest and violence as the canonical forms of contentious politics^4^, together with music events as the subcultural infrastructure that recruits members and sustains collective identity^3^. These three manifestations are at once central to the far-right repertoire and systematically documented at the county level in parliamentary and civil-society sources. However, so far no existing dataset integrates them at the local level.

Existing empirical research operationalises far-right activism along largely separate axes: electoral studies measure vote shares and party organisation; protest-event analyses and conflict-event databases record demonstrations and, less often, violence; and dedicated hate-crime or anti-refugee datasets capture violence in isolation^5,6^. Each investigates a single dimension, and none jointly tracks the observable forms through which the movement reproduces itself locally. Most databases exclusively focus on protest events^5,7^, neglecting the music scenes and localized violence that sustain extremist networks^3^. Existing resources on German far-right activism cover only part of this landscape. The Kanol *et al*.^8^ protest database, derived from parliamentary inquiries, provides the most comprehensive event-level record of far-right protests but excludes violence and music events. Benček and Strasheim^6^ as well as Jäckle and König^9^ offer georeferenced datasets on anti-refugee violence, but both are restricted to a single motive category. Hellmeier and Vüllers^10^ document Pegida protest mobilization across German cities from 2014 to 2017, yet their scope is limited to Pegida alone. The ACLED dataset covers political violence events in Germany but does not differentiate far-right motive subcategories at the level required for social-scientific analysis^5^. While the C-REX dataset includes these subcategories, it is limited to cases of severe and lethal violence^11^. We do not cover electoral politics, which dedicated resources such as the German Election Database already serve^12^, or manifold everyday and cultural practices such as grassroots welfare and community events^2^.

We address these gaps by introducing the Far-Right Activism (FARAC) dataset, a standardized, county-level database of German far-right activism covering the period from 2013 to 2024. Germany is well suited to this data consolidation because the federal government discloses intelligence on far-right music, protests, and violence in response to regular parliamentary inquiries^13^. By integrating these official disclosures with victim-led reporting from counseling centers, the dataset provides a complementary and potentially broader measure of violent acts than government statistics alone. Local protest event analyses have shown that national-level data systematically underrepresent local mobilization dynamics^14^, motivating our use of the county (*Landkreis*) as the primary unit of analysis. This choice also reduces biases from inconsistent sub-county information, as government responses usually do not give information as to the city district for cases in major cities such as Berlin or Hamburg.

**Fig. 1:**
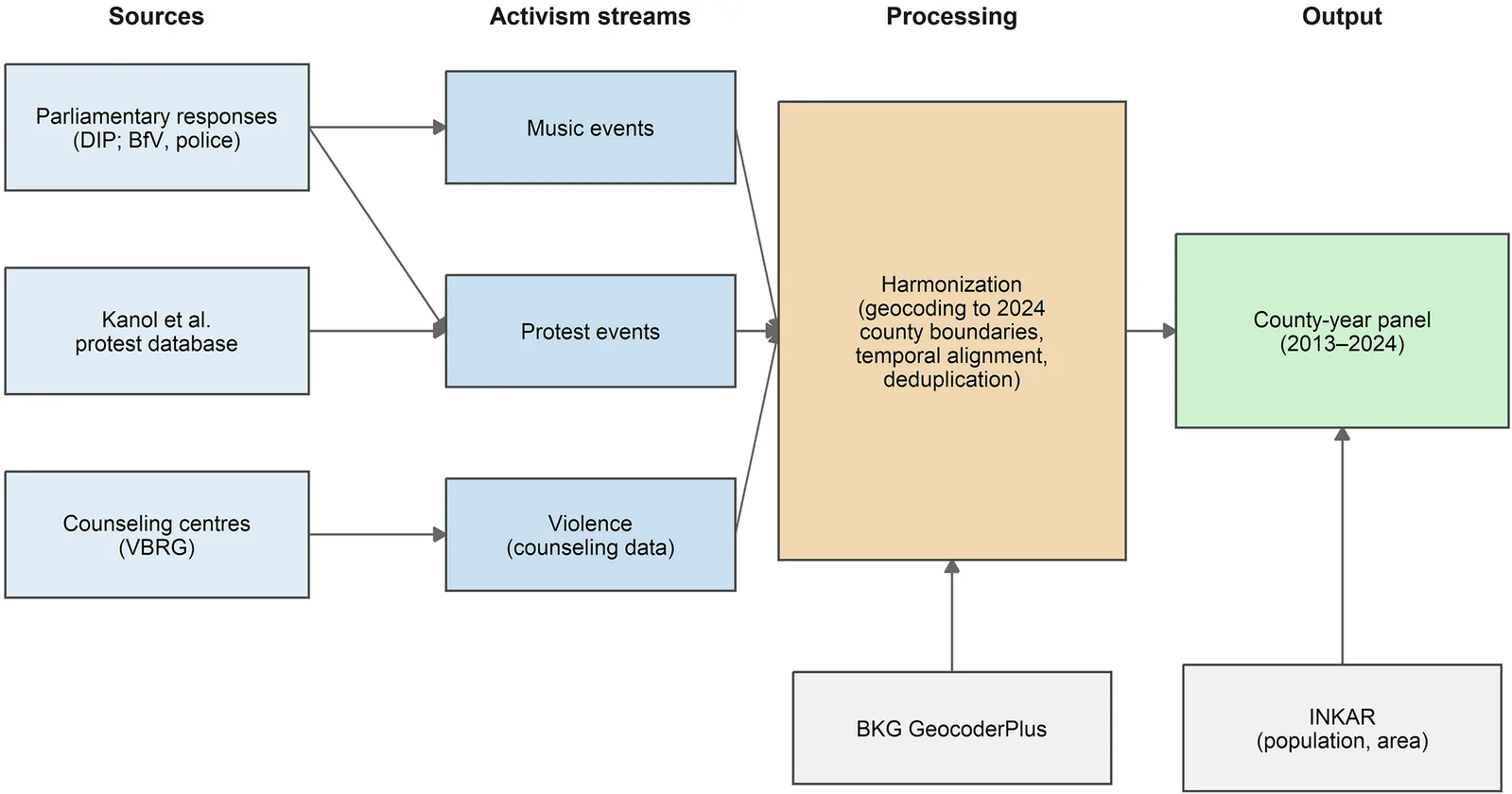
Overview of the data pipeline: input sources (left) feed three activism streams, which are harmonized to 2024 county boundaries and integrated into the county-year panel. BKG GeocoderPlus supports the harmonization step; INKAR supplies population and area covariates for the panel.

The resulting dataset introduces three technical innovations to the longitudinal study of far-right activism. First, it catalogues music events. Far-right music functions as movement infrastructure: music events in “white power free spaces” recruit members, finance activity, and sustain the collective identity of extremist networks^3,15,16^. Second, it aligns all protest data with 2024 county boundaries to maintain spatial consistency over the full panel period. Third, it expands the scope of far-right violence beyond anti-refugee incidents by incorporating the full range of motive categories documented by counseling centers^17^. These localized, multi-dimensional metrics support precise analyses of movement dynamics and provide a benchmark for validating automated protest event-coding systems that rely on news media^18^. Figure 1 summarizes how the input sources map onto the three activism streams, the harmonization step, and the resulting county-year panel.

## Methods

The dataset integrates three data streams at the county level: music events from government responses to parliamentary inquiries, protests from academic records and government responses, and violence documented by counseling centers. The dataset also includes alternative violence measures from government records to facilitate empirical alignment. A unified geocoding protocol standardizes these disparate event types across shifting administrative boundaries to maintain longitudinal consistency.

### Data sources and provenance

The music and protest event records derive from written government responses to parliamentary inquiries, retrieved from the German Bundestag’s Documentation and Information System (DIP; https://dip.bundestag.de). We located the relevant responses by searching DIP, filtered by electoral term (*Wahlperiode*), for the recurring quarterly inquiry titles: “Musikveranstaltungen der extremen Rechten im [N]. Quartal [Jahr]” for music and “Rechtsextreme Aufmärsche im [N]. Quartal [Jahr]” for protests (for example, https://dip.bundestag.de/suche?term=MusikveranstaltungenderextremenRechtenimerstenQuartal2023&f.wahlperiode=21); the *Drucksache* identifier of every response is recorded in the event-level sources field. The protest series additionally incorporates, for 2005–2022, the far-right protest database of Kanol *et al*.^8^ (GESIS, 10.7802/2774). County-level violence statistics are compiled by the counseling centres of the VBRG association; the contributing organisations and their coverage periods are listed in Table 1, the centres’ aggregate figures are published at https://verband-brg.de/archiv/zahlen-fakten/, and the URL of each underlying report is retained in the released violence file. Population and area covariates are drawn from INKAR (Laufende Raumbeobachtung des BBSR—INKAR, Ausgabe 07/2025; Bundesinstitut für Bau-, Stadt- und Raumforschung, Bonn; https://www.inkar.de)^19,20^. Geocoding used the BKG GeocoderPlus^21^.

**Table 1:** Counseling centers contributing county-level violence data, by state and coverage period.

Organization	State	Year Start	Year End
Opferperspektive	Brandenburg	2002	2024
Mobiles Beratungsteam	Saxony-Anhalt	2003	2024
RAA Sachsen	Saxony	2008	2024
LOBBI	Mecklenburg-Vorpommern	2013	2024
ezra	Thuringia	2015	2024
ReachOut/VBRG	Berlin	2015	2024
ZEBRA	Schleswig-Holstein	2018	2024
empower	Hamburg	2022	2024
OBR Rheinland	North Rhine-Westphalia	2024	2024

Each input is either a public official work or is licensed for re-use with attribution, and we use them within those terms. The Bundestag responses are official parliamentary documents (*amtliche Werke*), which are exempt from copyright under German law (§5 UrhG) and freely reproducible; the Kanol *et al*. protest database is distributed by GESIS under a Creative Commons Attribution (CC-BY) licence; the violence figures are the VBRG counseling centres’ published aggregate statistics, reproduced here with attribution to the contributing organisations (Table 1); the INKAR covariates are published under the Data Licence Germany – Attribution 2.0 (*Datenlizenz Deutschland – Namensnennung 2.0*, dl-de/by-2-0); and geocoding to 2024 counties used the BKG GeocoderPlus, attributed to its federal geobasis data (© GeoBasis-DE/BKG). Under the BKG terms of use (V GeoBund 2024), the point coordinates returned by the geocoder serve internal county assignment only and are not redistributed; the released event files therefore contain the county assignment, not the underlying coordinates, which any user can reproduce from the released location fields. No input carries personal data or confidentiality restrictions at the aggregation we release (see the Methods on the removal of personal identifiers). The dataset generated in this work is released under a CC-BY 4.0 licence, consistent with the terms of the reused protest source.

#### Music Event Data

Government responses to parliamentary inquiries regarding far-right music events are archived in the German Bundestag’s Documentation and Information System (DIP)^22^. These inquiries take the form of recurring written parliamentary questions (*Kleine Anfragen*), submitted predominantly by the parliamentary group of the Left party (*Die Linke*) on a roughly quarterly basis, to which the federal government replies in writing. Each reply is published as a numbered Bundestag printed paper (*Drucksache*)^13^. We queried the DIP for “government responses” concerning these events up to late 2024 to construct the primary dataset. The federal government compiles these responses on the basis of information from the security authorities, principally the domestic intelligence services (the federal and *Länder* Offices for the Protection of the Constitution, BfV/LfV), which monitor the far right through open sources and confidential human sources. Each response lists event dates, locations, and performing artists. As agencies frequently update counts in subsequent quarters, our dataset links single events to multiple document IDs to ensure a continuous record. We record the document numbers (*Drucksachennummern*) of all underlying government responses in the event-level sources field (format Drucksache <Wahlperiode>/<no.>, e.g. Drucksache 18/13256; multiple identifiers are semicolon-separated and can be extracted with the pattern Drucksache \d+/\d+), so that every event can be traced back to its source documents and the complete list of underlying inquiries ships with the data. County-level granularity is otherwise absent from the official annual LfV reports, which provide only state-level aggregate totals and thus obscure local variance.

**Fig. 2:**
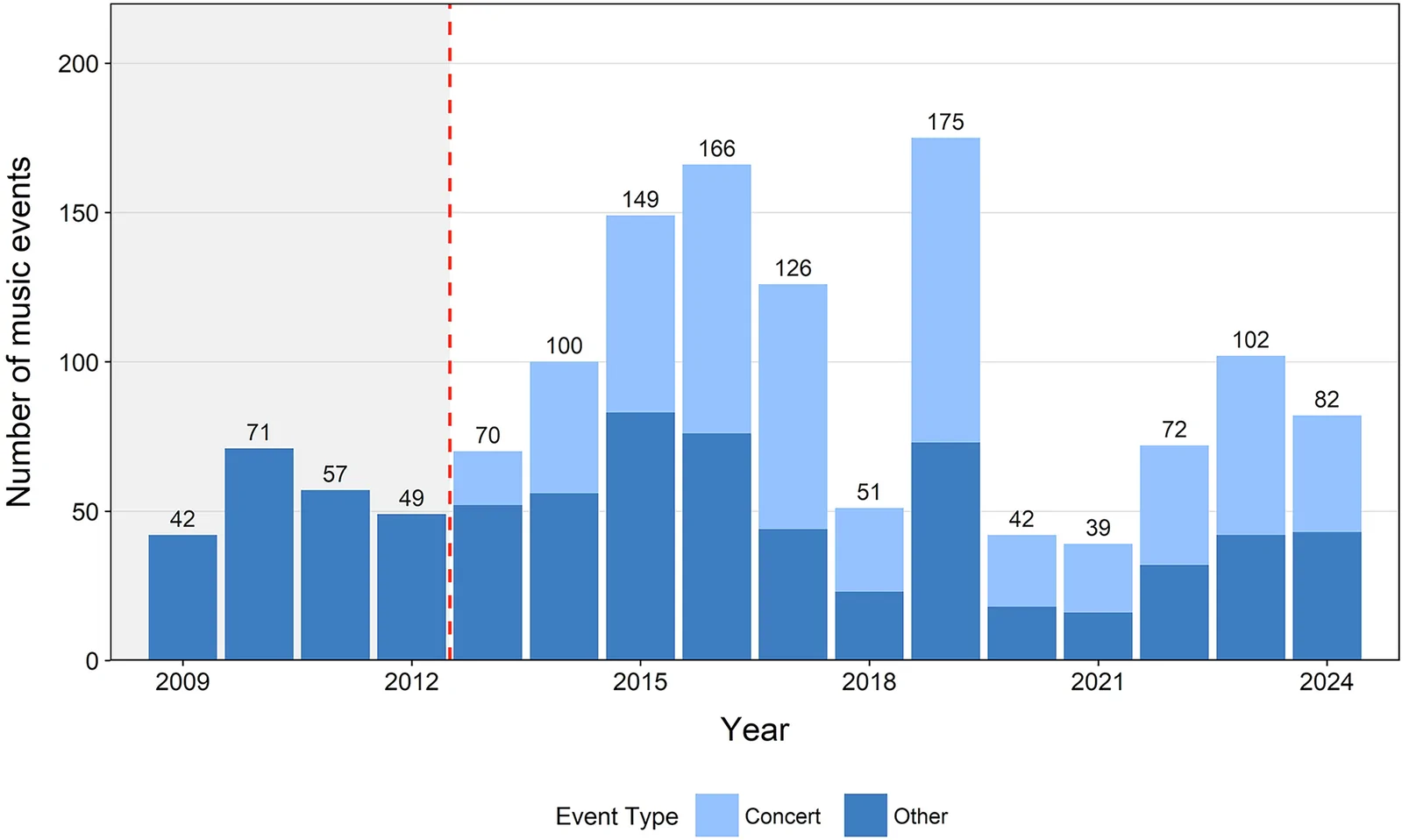
Annual frequency of far-right music events in Germany, 2009–2024. The harmonized panel begins in 2013, the first year of complete music coverage (concerts are reported only from 2013); shaded earlier years predate the panel and are shown to document the full extent of the source data.

These administrative records allow for a clear distinction between “concerts” and “other music events” based on the primacy of the musical performance. “Concerts” feature far-right performers as the focal point, whereas “other events” relegate music to a secondary role within political or social gatherings. Each record contains a date, event type (concert or other music event), location, state, and performing artists. Figure 2 illustrates the annual frequency of these events across the full period available in the DIP. Concert records first appear in the government responses in 2013; earlier responses document only other music events. Because complete music coverage begins in 2013, that year sets the start of the harmonized county-year panel: protests are recorded from 2005 and could in principle extend the series to an earlier starting point, but the music stream is the binding constraint on joint coverage. The pre-2013 years shown in Fig. 2 therefore document the full extent of the source data rather than forming part of the panel. The responses are explicit about their limitations: only events that were openly announced or held are listed individually with date and location, whereas covertly organized (“conspiratorial”) events known to the authorities appear only in aggregate counts (the Q4 2024 response, for example, lists 22 openly held events alongside 19 covertly organized ones; *Drucksache* 20/14657), and some details are withheld to protect the authorities’ state of knowledge and their confidential sources. However, even these aggregate totals capture only what the authorities themselves have detected, so the actual frequency of far-right music activity is considerably higher than any publicly available source can document.

To transform these qualitative reports into structured data, we extracted the tabulated entries directly from the government-response PDFs using the tabulapdf package, which reads the documents’ native text layer; no Optical Character Recognition (OCR) was required. Where information was not tabulated but appeared only in the running text of a response, we collected it manually, capturing low-frequency events and minor late reports that fall outside the structured tables. We then geocoded these events with the BKG GeocoderPlus^21^ and assigned them to 2024 administrative divisions; the geocoding refinement and the rationale for county-level aggregation, common to all streams, are described under Comparability and Harmonization.

This spatial standardization allowed for the final identification and merging of duplicate entries of the same event, as indicated by the same location and date. Manual inspection confirmed these duplicates represented overlapping reports rather than simultaneous performances. Later government responses often provided supplemental details, such as revised band lists or organizer names, which we consolidated into single event series. This deduplication reduced the final event count relative to the raw number of entries in the source documents.

#### Protest Events Data

We adopt the protest database compiled by Kanol *et al*.^8^, which draws on the same type of government responses to parliamentary inquiries in the Bundestag (quarterly recurring *Kleine Anfragen*, again predominantly from the Left party, *Die Linke*) and covers far-right demonstrations from 2005 to 2022, including event dates, locations, participant counts, and protest mottos. We expanded these records to cover the 2023–2024 period using the same source type, and geocoded every entry in the combined dataset through BKG GeocoderPlus. As with the music data, the originating *Drucksache* identifiers are recorded in the event-level sources field (e.g. Drucksache 17/2804). Because participant counts in this source reflect government estimates rather than independent verification, they should be treated as approximate. The protest responses are limited to activities recorded by the authorities. Where events are listed, the reported figure states the estimated total attendance; the responses note explicitly that the number of far-right participants may differ from this total (e.g., *Drucksache* 20/14656). While late reporting may necessitate minor updates to recent entries, this foundation allows for systematic subnational comparisons.

The inclusion of participant counts adds analytical value that justifies the additional technical effort^23^. These counts distinguish the protest records from music events data, where authorities withhold attendance figures, citing security concerns. We are able to leverage these participant numbers to calculate annual totals, minimums, maximums, and means for each county. Such metrics capture protest intensity more accurately than simple event counts, and high-intensity events generate distinct socio-political consequences.

Capturing participant numbers accurately requires specific rules for multi-location protests on a single day^24^. We treat events confined to one county as single-location protests with aggregated totals. For events spanning multiple counties, we distribute the total participant count equally across all listed locations. Specific indicators in the final dataset allow researchers to isolate or exclude these distributed protests for the purpose of analysis. Researchers interested in protest episodes rather than individual protest events can construct episodes using these indicators alongside the protest mottos provided in the event-level data.

Beyond managing spatial distribution, we deduplicate entries sharing the same date and location to ensure unique event counts^24^. Most duplicate entries represent administrative updates, such as revised participant counts, rather than parallel events. We calculate all participant metrics (mean, min, max) before county-level aggregation to consolidate these partial observations. Qualitative details that resist numerical aggregation are merged into the final event rows to preserve descriptive detail.

**Fig. 3:**
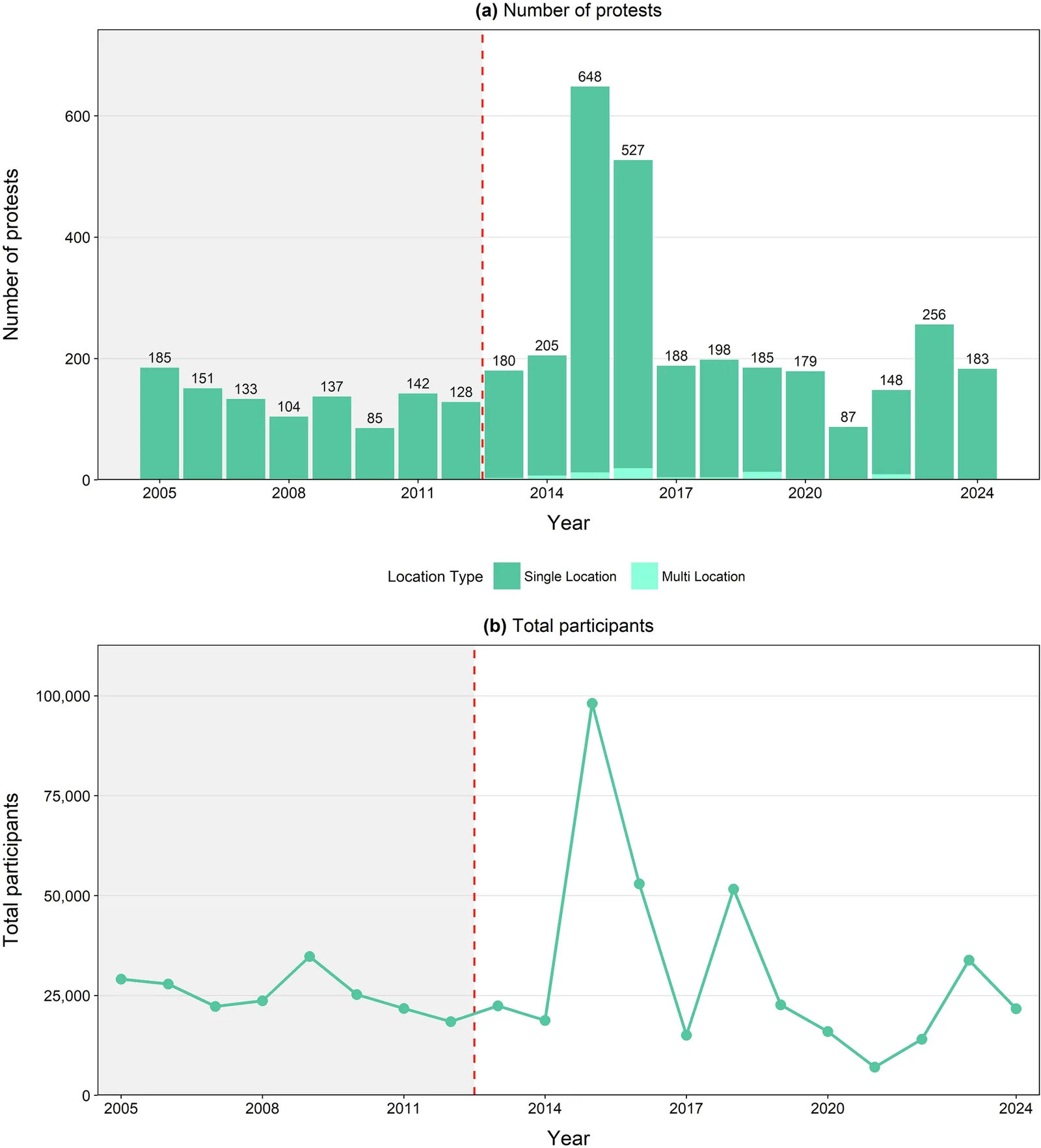
Annual frequency and total participants of far-right protests in Germany, 2005–2024. (**a**) Number of protests by location type (single vs. multi-location). (**b**) Germany-wide total participants per year. Shaded years before 2013 predate the harmonized county-year panel and are shown to document the full extent of the protest source data.

This merging and cleaning process mitigates processing artefacts such as duplicate records and inconsistent identifiers, though it cannot remove biases originating in the sources themselves. As illustrated in Fig. 3, single-location protests constitute the vast majority of events, and the deduplication process resulted in only a marginal reduction in total event counts. The observed divergence between trends in event frequency and participant numbers confirms the necessity of including both metrics to represent protest dynamics. These patterns are consistent with the data’s longitudinal use; researchers should nonetheless make the modelling choices appropriate to their own questions.

#### Violent Events Data

Independent counseling centers (*Beratungsstellen für Betroffene rechter, rassistischer und antisemitischer Gewalt*) provide the county-level statistics for far-right violence. These centers exclusively record violent offenses (*Gewalttaten*): assault, dangerous and grievous bodily harm, arson, homicide, coercion, credible threats with severe consequences, and massive property damage directed at individuals. They exclude verbal insults, propaganda offenses, and minor property damage without a personal target^17^. Centers capture incidents not included in official security records because victims frequently avoid police contact. Information reaches the centers through victims and their communities, local cooperation networks, verified police reports, press coverage, and monthly parliamentary inquiries on politically motivated crime (PMK) in state legislatures. A case enters the statistics only when sufficient verified information permits classification according to the centers’ standardized criteria; hearsay reports are excluded. This documentation process relies on a sustained local presence, which creates regional variance in data availability across German states. While East German states like Saxony maintain continuous records dating back to the 2000s, newer centers in states such as Hamburg and North Rhine-Westphalia began publishing only recently (2022 and 2024, respectively). Counseling centers now operate in every federal state, but only a subset publish figures at the county level; the remainder report only aggregate or state-level totals, or do not publish any numbers at all.

The umbrella association of counseling centers (VBRG) coordinates a standardized monitoring framework that harmonizes counting rules, motive categories, offense type classifications, and severity thresholds across all member organizations. These shared quality standards align the centers’ definition of politically motivated violence with the federal police definition of politically motivated crime (*Politisch motivierte Kriminalität*, PMK), without being identical to it. The PMK system was established by the Federal Criminal Police Office in 2001 and most recently revised in October 2024^17^. While the PMK system requires evidence of political motivation at the time of police recording, counseling centers classify incidents on the basis of the victim’s perception and situational indicators, yielding systematically higher counts. This standardization has progressively improved cross-regional comparability, though records prior to each center’s adoption of the common framework may reflect organization-specific classification practices.

**Fig. 4:**
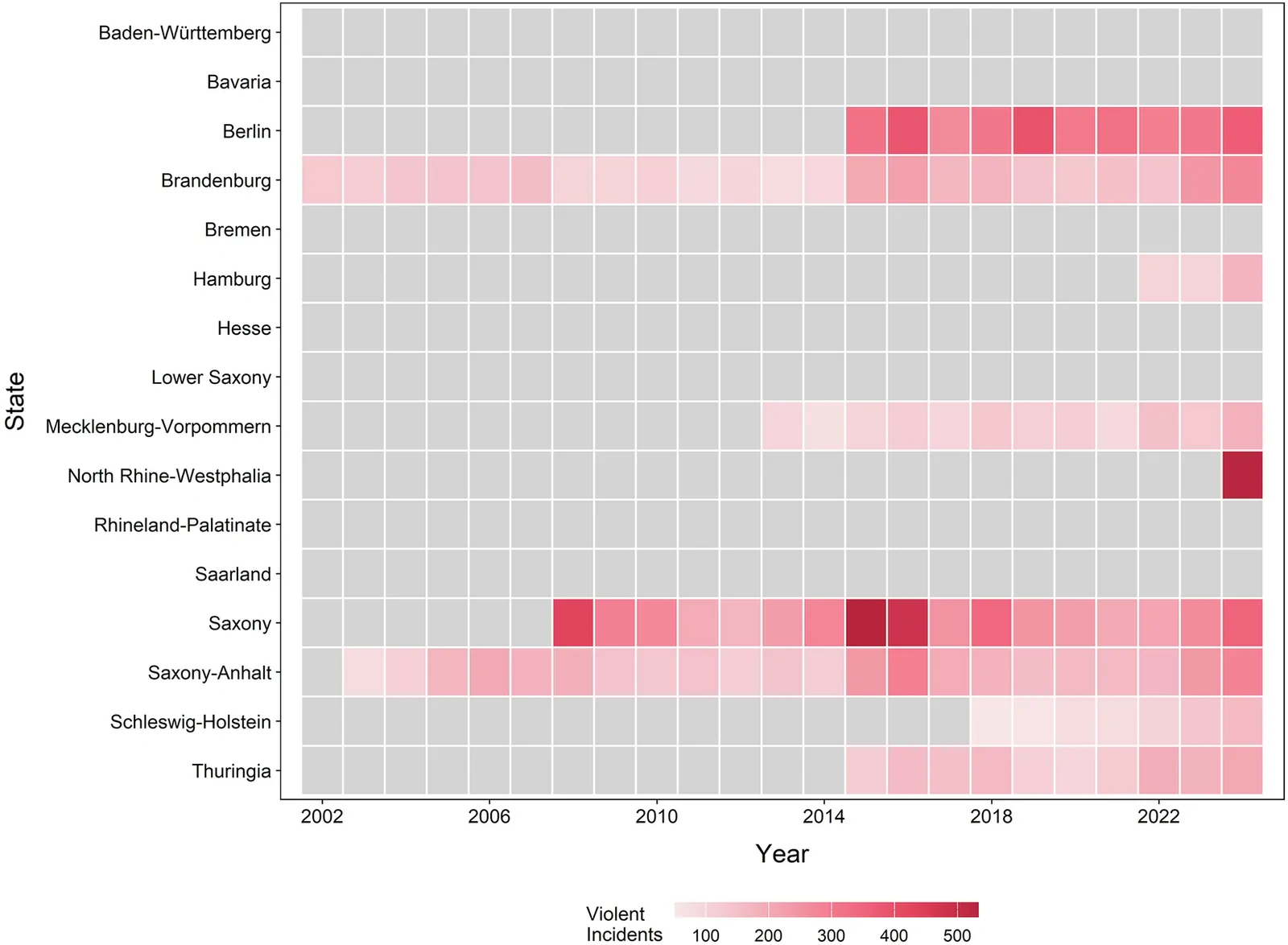
Geographic and temporal coverage of county-level counseling center violence data.

Figure 4 maps the geographic distribution of these established counseling centers and the temporal reach of their respective datasets, while Table 1 lists each contributing organization alongside its state and coverage period.

Visible reporting gaps are not random but stem from divergent civil-society activity. Counseling centers were established early and with the most extensive scope of operation in East German states in response to the high levels of violence recorded in the 1990s. We accordingly excluded several West German states (Bavaria, Baden-Württemberg, Hesse, Rhineland-Palatinate, Lower Saxony, Bremen, and Saarland) where counseling centers either lack the capacity for systematic county-level monitoring or publish only selected cases in their chronologies, which omit the verified case-level detail that the VBRG quality standards require for inclusion^17^. Because the absence of data in these regions reflects institutional histories rather than a zero-count of violence, standard listwise deletion or mean imputation remains inadvisable. Researchers should instead model the probability of observation alongside the violence counts. We include a violence coverage variable in the final dataset to facilitate this requirement.

Consistent county-level units support rigorous statistical analysis of temporal and regional variance. Multilevel models can control for systematic differences across states and time. To support this approach, the dataset includes explicit coverage flags and organizational identifiers for each source. We recommend utilizing the most recent annual figures for cross-sectional studies, while longitudinal analyses require multilevel modeling to account for staggered standardization across centers.

The categorical scope of counseling center records is an advantage over alternative sources. Unlike studies limited to anti-refugee attacks^6,9^, these records classify each incident by a detailed set of perpetrator motives: racism (further differentiated into anti-Black, anti-Asian, anti-Slavic, and anti-Muslim racism), antisemitism, anti-Roma hostility, hostility based on sexual orientation or gender identity, social-Darwinist motives (targeting homeless people and people with disabilities), and attacks on political opponents including journalists, elected officials, and civil-society activists^17^. The classification follows both perpetrator indicators (utterances, clothing, organizational affiliation) and situational criteria (pattern of repeated attacks, victim selection), with the victim’s perspective serving as the primary criterion for assessing the circumstances of the attack. Because counseling centers do not publish category-specific counts at the county level, this dataset provides total incident counts per county and year, aggregated across all motive categories, rather than disaggregated breakdowns by perpetrator motive. This broader count nonetheless improves on sources restricted to a single motive category, consolidating these independent datasets at the county level for the first time.

#### Comparability and Harmonization

Shifting administrative boundaries impede the aggregation of longitudinal data on German music, protests, and violence. Between 2013 and 2024, administrative mergers reduced the total number of counties from 405 to 400. Despite the small number of these administrative changes, they create inconsistent spatial units, which complicates temporal comparisons and risks aggregation bias. Valid longitudinal analysis therefore requires systematic unit harmonization.

We resolve these inconsistencies by mapping every record to a fixed 2024 county map. We assign all music and protest events to their 2024 counties from their geocoded coordinates (using the BKG GeocoderPlus), independently of the historical names in the source, and we geocode the county names reported in counseling-center statistics to the corresponding 2024 jurisdictions. Where boundary reforms merged multiple historical counties, we sum the affected incidents within the new 2024 perimeters. We then aggregate all variables by county and year, calculating missingness variables for participant data where necessary. This uniform spatial assignment integrates the fragmented datasets into a single coordinate system.

Government responses contain location data that are consistent but sometimes factually inaccurate. For example, authorities frequently misassign locations to federal states. We resolved these errors through an iterative process using the BKG GeocoderPlus. First, we geocoded locations without state boundaries; then, we manually adjusted discrepancies, such as correcting “Karow” to “Karow bei Genthin.” When the geocoder failed to identify a town within a reported state, we integrated the tool’s alternative coordinate suggestions. Because many German place names are non-unique, we disambiguated same-named towns using the federal state reported with each event: the state identifies which place is intended; for example, “Freiberg” resolves to *Freiberg* in Saxony or to *Freiberg am Neckar* in Baden-Württemberg, and “Frankfurt” to *Frankfurt am Main* in Hesse or *Frankfurt (Oder)* in Brandenburg. Where the reported state was itself inaccurate, the iterative correction described above recovered the intended location; the few genuinely unresolvable or cross-border cases were coded as missing. To preserve data integrity, the dataset maintains separate variables for original and cleaned data (location_original vs. location_clean). The location_was_corrected flag allows researchers to audit every modification.

We geocoded all entries by prioritizing the county over sub-county levels. Because data below the county level are unavailable, and major cities often omit neighborhood specifics, we aggregate data only at the county level. Areal interpolation of urban data based on population density would introduce unacceptable imprecision. This county-level focus reconciles the dataset with counseling center violence data, which exists only at that scale.

**Fig. 5:**
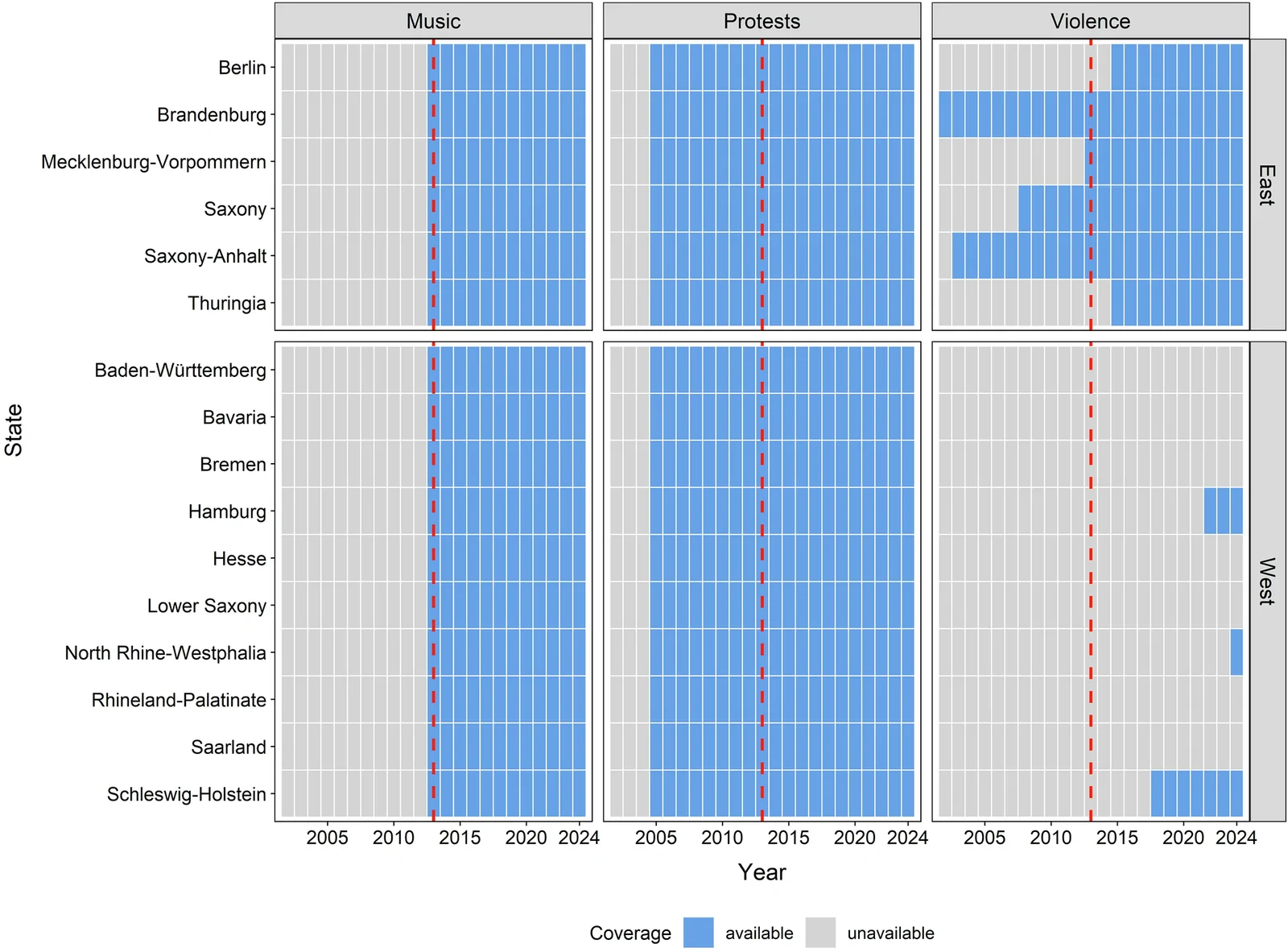
Data availability for music, protest, and violence by state and year. The dashed red line marks 2013, the start of the harmonized panel.

Figure 5 delineates the specific states and years for which county-level coverage of music, protest, and violence is available. The panel begins in 2013, the first year for which the music stream is completely covered (concert reporting starts then); protests extend back to 2005, so the music stream is the binding constraint on the joint start year. Counseling-center coverage of violence, by contrast, expands over time, from a handful of eastern states in the early years to a broader set from 2015 onward, so violence is observed for fewer counties in 2013 and 2014 than later in the series. We retain these early years rather than discard otherwise complete music and protest data, and we flag the sparser early violence coverage explicitly so that users can restrict the panel to higher-coverage years if their analysis requires it. The framework distinguishes between structural “missing” values (where no counseling center coverage existed) and “zero” values (where coverage existed but centers recorded no events). Although the primary dataset uses 2024 boundaries to ensure temporal consistency, researchers may revert to historical boundaries using crossfiles from the Federal Institute for Research on Building, Urban Affairs and Spatial Development (BBSR).

All three streams are jointly available from 2013 onward, the first year the music stream is completely covered, though institutional reporting standards restrict the geographic coverage of far-right violence, particularly in the early years (see the Violent Events Data section). County-level aggregation further obscures within-county spatial variation; researchers requiring finer resolution should consult the event-level files, though these remain subject to the same large-city resolution limits described above.

Harmonization does not preclude independent analyses of specific activism types or long-term regional studies. Instead, it centralizes previously fragmented event datasets so that researchers can pursue a range of designs. With a unified administrative framework, researchers can investigate individual variables or the intersections between music, protest, and violence.

## Data Records

All data records are deposited as the Far-Right Activism (FARAC) dataset on Zenodo^25^ (https://doi.org/10.5281/zenodo.21041488). This repository includes raw data for music events and protests, along with a dataset that harmonizes all three activism types. The resulting balanced panel comprises 400 counties observed over 12 years (2013–2024), yielding 4,800 county-year observations. Although the panel is balanced by construction, the violence stream is observed only where and when a counseling center was active: of these 4,800 county-year cells, civil-society violence data are available for 1,034 (21.5%): 986 recording at least one incident and 48 an observed zero, while the remaining 3,766 cells are missing rather than zero. Because coverage begins in different years by state (Table 1), the aggregated violence_county_year file comprises 1,434 county-year rows spanning 2002–2024 (1,031 within the 2013–2024 panel window). The balanced panel should thus be read as nominal coverage, with violence treated as missing outside covered state-years. Violence coverage is also thinner in the earliest panel years (2013–2014), when fewer counseling centers reported county-level data; these years are retained, with violence coded as missing where no center was active. To maintain spatial consistency, these harmonized data utilize 2024 county boundaries; the repository further provides a corresponding GeoPackage file with 2024 county boundaries. Population (residents per county) and area estimates (km^2^) are included in the harmonized panel as annual variables covering 2013–2024, sourced from INKAR^19,20^; INKAR reports these figures on the same fixed 2024 county definitions used throughout, so they require no separate boundary harmonization and are matched to the panel by county identifier (AGS). As INKAR data are typically published with a one-year lag, the 2023 population and area figures are carried forward to 2024 (county areas change only with boundary mergers, the last of which predates the panel’s final years). All files use.csv and GeoPackage formats, with Table 2 detailing specific metadata, names, and timeframes. Detailed codebooks describing all variables for each dataset are provided alongside the data files in the repository.

**Table 2:** Inventory of the FARAC datasets, including format, time period, geographic unit, and row count.

Dataset Name	Type	Time Period	Geographic Unit	N (rows)
far_right_activism_panel	harmonized panel	2013–2024	county (2024 boundaries)	4,800
music_events	event-level	2009–2024	county (2024 boundaries)	1,545
protest_events	event-level	2005–2024	county (2024 boundaries)	4,052
music_protest_monthly	aggregated	2013–2024	county-month (2024 boundaries)	2,705
violence_county_year	aggregated	2002–2024	county-year	4,052
official_violence	official data	2015–2024	state-year	160
county_boundaries_2024	GeoPackage	2024	county	400

Row counts reflect file size. For violence_county_year, county-years are observed only under counseling-center coverage: within the 2013–2024 panel, civil-society violence data cover 1,034 of 4,800 county-year cells (21.5%); the remainder are missing rather than zero.

## Technical Validation

We validate each processing step (digitization, geocoding, deduplication, and boundary assignment) against source documents and external benchmarks. Our tests identified technical errors introduced during location cleaning and date standardization, while confirming that the underlying text extraction, performed on the PDFs’ native text layer without OCR, was accurate. We also detected duplicate event entries and made sure that administrative divisions match their spatial assignments. This systematic verification removed the processing artifacts described in the following sections.

### Data Integrity and Extraction Quality

The tabulapdf R package^26^ extracted the native text layers embedded within the federal government PDFs. To verify extraction quality, we compared a 1% manual sample of music and protest events against these source files. This comparison yielded error rates of 0.0% for location and date fields. Such high accuracy stems from the native text layers within the source documents; no Optical Character Recognition was required. These tests confirm that extraction succeeded for both the original entries and the integrated Kanol *et al*.^8^ dataset.

Reliable extraction of text layers does not ensure that the original authorities provided error-free entries. To address misspellings and inconsistencies, we removed control characters, normalized whitespace, and iteratively standardized location and state names. We replaced imprecise official entries (e.g., “Braunschweig area”) with specific city or municipality names to assign each event to a unique administrative division. When a location crossed borders or the authority data was missing, we coded the entry as NA. This coding preserves the raw data for researchers studying individual events, even when spatial aggregation requires their exclusion. Consequently, we assigned NA status to 9.8% of music events and 0% of protest events.

In contrast, the violence dataset required no NA assignments for imprecise locations. We only had to attribute 10 violent acts to the state level rather than to specific counties.

### Inter-coder Reliability

The checks above were performed by the same team that built the extraction and geocoding pipeline. To assess coding reliability, the coauthor (Mayer), who had not participated in the original extraction or geocoding, re-coded a random sample of events directly from the source documents while blind to the released data files and the original coding. We drew a reproducible (seed 20260619), stratified random sample of 351 events (176 music events across all years and 175 protest events from the author-coded 2023–2024 extension), allocating draws proportionally across event-type-by-year strata. The 2005–2022 protest records adopted from Kanol *et al*.^8^ were not re-coded, since their reliability is established in that source.

Working solely from the Bundestag *Drucksachen*, the second coder re-derived each event’s type, administrative division, and (for protests) participant count, following a written coding manual. Crucially, the county was assigned independently of our pipeline: the coder resolved each place name to its 2024 county using the official Regionalatlas^27^ rather than the BKG geocoder used in the pipeline, so that agreement on the county reflects genuine convergence rather than a shared tool. We then computed inter-coder agreement against the released values, using Cohen’s κ and Krippendorff’s α for the categorical fields (event type, county) and the intraclass correlation coefficient for participant counts.

Agreement was high across all fields: event type (99.1% agreement, κ = 0.99, α = 0.99), county assignment (95.8% agreement, κ = 0.96, α = 0.96), and protest participant counts (ICC = 0.95, with an exact match in 96.9% of cases). We adjudicated every disagreement against the source document. Of the 17 disagreements, 5 reflected genuine ambiguity in the original government response: non-unique place names (for example, several distinct localities named *Schönow* in Brandenburg, or vague region descriptors such as “Raum Karlsruhe” (the Karlsruhe area) that can refer to both a city and its surrounding district) and two borderline event-type classifications. The remaining 12 were clerical or definitional discrepancies on the coder’s side (such as a mis-keyed county code) that were resolved in favor of the released values; none revealed an error in the released data. This re-coding confirmed that the sampled records can be reproduced from the source documents by a second coder who had not participated in the original extraction or geocoding.

### Temporal Consistency and Date Adjustments

Government responses contain temporal inconsistencies that mirror errors in the location data. While most music events and protests are linked to specific dates, imprecise entries frequently stall automated processing. To standardize these records, we mapped multi-day periods to the first mentioned date. For entries reporting only a month, we defaulted to the first day. Because the violence dataset lacks precise timestamps, we aggregate county-level data only by year. To assist granular analysis, we provided quality flags (exact, month, year, missing) for precision-based filtering. Ultimately, incomplete information forced us to code 16 music events (1%) and 2 protests (0%) as “NA,” while manual adjustments corrected 66 music events (4.3%) and 0 protests (0%). Because no event in the original records spans an annual boundary, the dataset maintains temporal integrity for time-series analyses.

### Relational Integrity and Deduplication

Because official government reports often contain inconsistent data, researchers must systematically deduplicate music events and protests appearing across multiple government responses. When entries were identical, we removed the duplicates; this process affected 8.4% of music events and 0.3% of protests in the raw dataset. Entries for the same date and location occasionally conflict regarding performers, organizers, or participant numbers. Because discrepancies usually represent updated records rather than distinct events, we merged all unique names into a single qualitative entry, retaining every name reported in any response. When quantitative data on protest participants conflict, researchers cannot reliably sum or average the figures. We therefore recorded the minimum, maximum, mean, and total sum of participants for each event. These multi-valued records allow researchers to test hypotheses across different participation scenarios and maintain transparency regarding data uncertainty.

### Geospatial Validation

We successfully geocoded 90.2% of music events and 100% of protests, assigning them to 2024 administrative divisions. Only a small fraction of violence data remains unassigned in cases where counseling centers reported broad monitoring areas. Spatial point pattern tests (SPPTs^28^) indicate that the spatial distribution of violence incidents remains highly stable year-over-year (r = 0.81–0.97), exceeding the 0.85 reference threshold in every year from 2015 onward. The single lower value in 2014 reflects the sparser counseling-center coverage of the early panel years and still indicates strong spatial correlation. Music events and protests show lower year-over-year spatial stability (music: r = 0.39 on average; protests: r = 0.6 on average). These lower correlations are consistent with genuine temporal shifts in far-right mobilization, but the tests cannot rule out contributions from changes in reporting, coding, classification, or geocoding. We therefore read them descriptively rather than as evidence of any single mechanism. To check that the 2024-boundary harmonization itself introduces no artefact, we compared year-over-year spatial stability in the counties affected by the administrative mergers (Göttingen, 2016; Wartburgkreis, 2021) against adjacent, non-merger years. Stability does not deteriorate in the merger years, consistent with the harmonization not introducing systematic spatial bias, though this comparison cannot fully exclude it. Researchers should nonetheless interpret event count increases in merger-affected counties as administrative artefacts rather than behavioral changes.

### Validation of Divergence

**Fig. 6:**
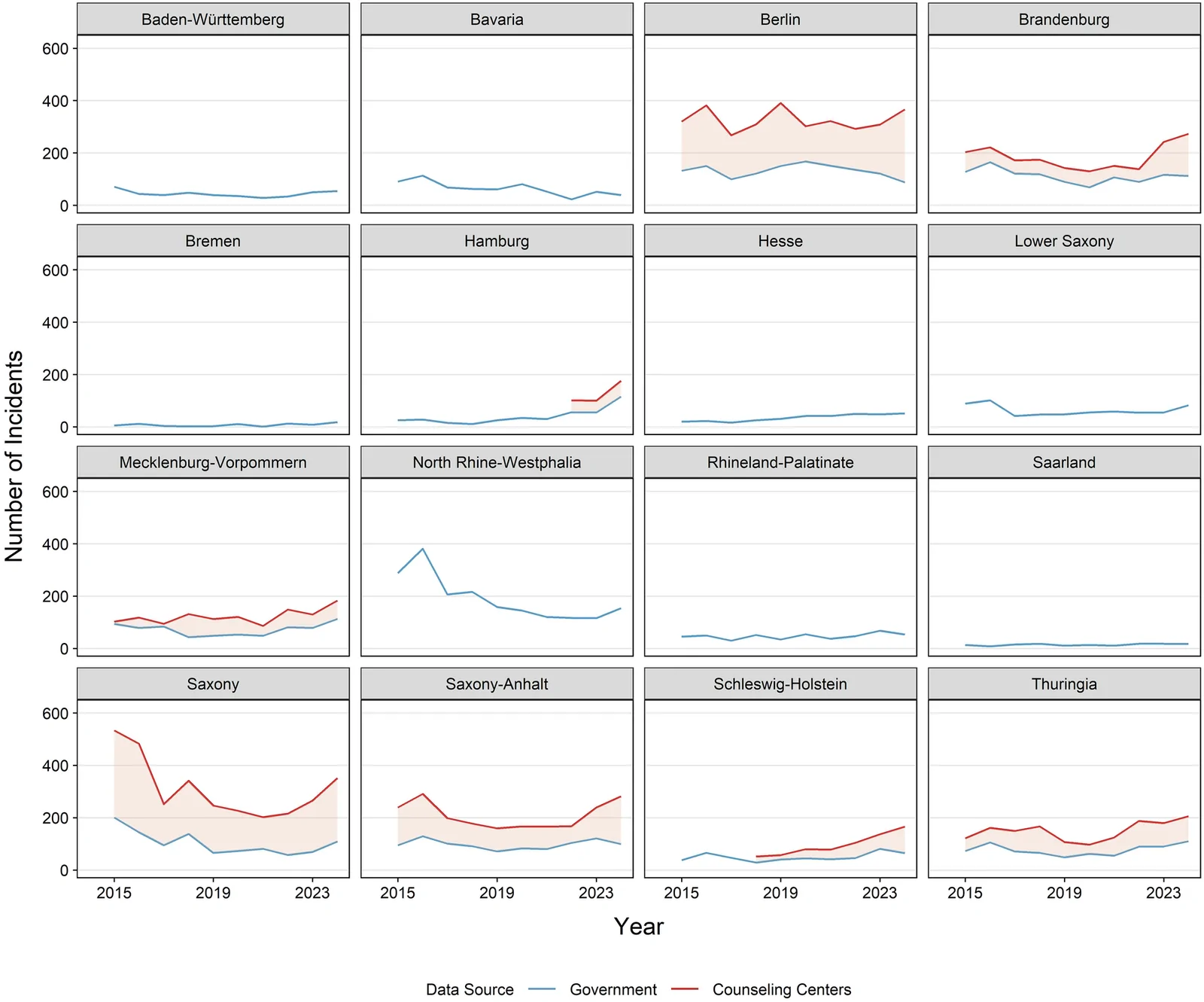
Comparison of government and counseling center violence counts by state, 2015–2024.

We validate counseling center data by comparing these figures against official government records across federal states from 2015 to 2024. Both sources covary strongly (r = 0.79; n = 71 state-year observations). This correlation indicates that while counseling centers consistently report higher absolute numbers than state agencies, both datasets track the same spatio-temporal patterns. The divergence between these sources reflects three systematic, substantively interpretable differences: (1) counseling centers count attacks, which may encompass multiple criminal offenses within a single incident, while the PMK system counts individual offenses; (2) police classification requires evidence of political motivation at the time of recording, whereas counseling centers later classify on the basis of the victim’s perception and situational indicators; and (3) victims who distrust law enforcement, particularly migrants and other marginalized groups, report to counseling centers but not to police, creating a structural dark field in official statistics^17^. That independent data sources on far-right and hate-motivated phenomena differ systematically in their definitions, recording thresholds, and reporting logics, rather than merely in accuracy, is well documented in the measurement literature^29,30^. As visualized in Fig. 6, the counseling centers’ elevated figures are hence best read as a substantive dark-field indicator rather than as a discrepancy in accuracy alone.

## Usage Notes

**Fig. 7:**
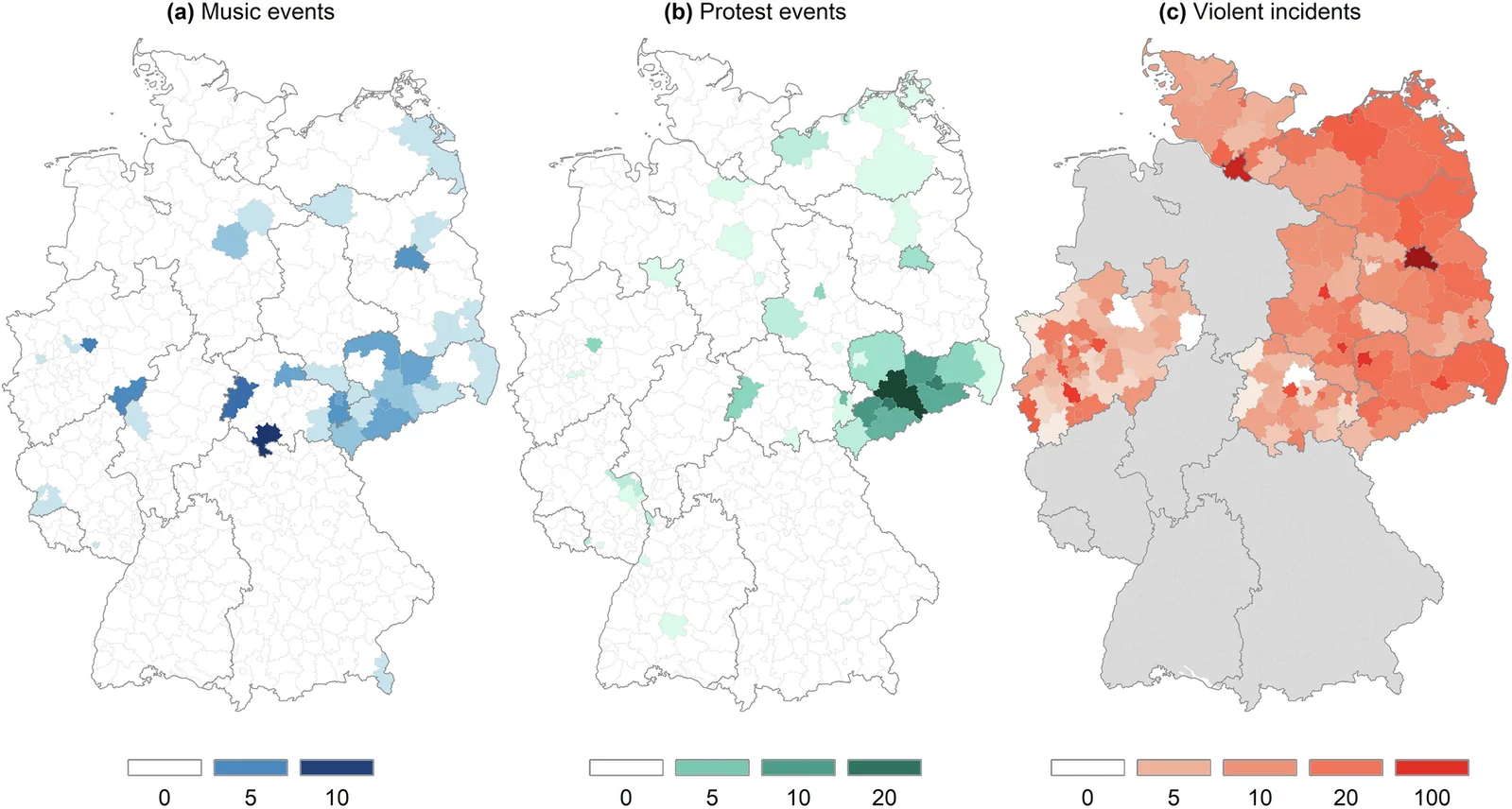
County-level distribution of far-right music events, protests, and violence in 2024 (absolute counts).

Figure 7 maps far-right music events, protests, and violent incidents across German counties in 2024. These subnational data reveal substantial internal variation which state- and nation-level aggregates often obscure^10,14^. While all three activism types concentrate in East German counties, neighboring administrative units often diverge sharply. Gray areas on the violence map signify missing data, not peaceful conditions. These gaps emerge because counseling centers, the primary data source, either lack a presence in certain counties or do not publish county-level statistics. Because this data gap primarily affects West German states (including Bavaria, Baden-Württemberg, Rhineland-Palatinate, Hesse, Lower Saxony, and Saarland), researchers should utilize the violence_coverage variable to prevent bias from listwise deletion. Music event counts remain considerably lower than official government reports because government responses exclude non-public events to protect state security. Because music events are frequently organized clandestinely and observed with varying capacity across states, the reported counts are minimal estimates. With events often being organized and attended by non-locals, a high count signals the movement’s organizational capacity rather than necessarily a local stronghold, while an absence of recorded events should not be read as an absence of far-right activity. More broadly, all three streams are institutionally selective measures: they capture far-right activism as documented by the recording institutions (parliamentary inquiries answered from the security authorities’ information on music events and protests, and civil-society counseling centres for violence) rather than a complete census. Each is therefore best read as a valuable but partial indicator whose per-stream limitations (covertly held music events and small local protests that go unlisted; counties without counseling-centre coverage) should guide interpretation and modelling. Because this institutional selectivity is itself non-random (counseling-centre coverage, for instance, is denser in some regions and political contexts than in others), the selection processes may correlate with covariates of substantive interest such as region, urbanity, or prior far-right strength. Analyses that condition on such variables should therefore treat coverage as potentially non-random rather than missing completely at random, and use the violence_coverage flag accordingly. While the dataset disaggregates multi-location protests by county (splitting the reported participant total equally across the listed counties), this equal split is a modelling convenience rather than an observed county-level measure; the group_event_id, county_list, n_counties, and single_location variables let users isolate individual events or reconstruct any alternative allocation rule. All records align with 2024 county boundaries, enabling direct integration with electoral results^12^, sociodemographic indicators, and other county-level datasets. Historical crosswalks further allow researchers to map these records to earlier administrative boundaries for longitudinal analysis.

Researchers combining these records should distinguish overlap that validates from overlap that double-counts. Within the panel, the three streams are conceptually distinct event types and are stored in separate columns (n_music_events, n_protests, n_violence_incidents); they are never summed into a single activity total, so cross-stream aggregation cannot inflate counts by construction. A single far-right gathering may nonetheless generate more than one record. A demonstration that turns violent, for instance, appears both as a protest and as a violent incident, but these are two measurements of different facets of the same event rather than duplicates, and should be retained as such. The clearest double-counting risk lies between the two violence files: violence_county_year.csv (counseling centres) and official_violence.csv (state security statistics) measure the same underlying phenomenon from independent sources and are intended for mutual cross-validation (see the Validation of Divergence above), not for addition; users should adopt one as their outcome rather than pooling both. The protest series is likewise an append rather than a merge: the adopted Kanol *et al*.^8^ records (2005–2022) and our extension (2023–2024) cover disjoint periods and do not overlap. Overlap between these data and external collections such as ACLED raises a separate question of cross-dataset positioning rather than internal double-counting.

Table 3 situates this dataset among the principal alternatives that, like ours, record far-right *acts* (events and incidents) rather than organisational presence. The German far-right-violence landscape is layered: official politically-motivated-crime statistics; the media-based *Chronik flüchtlingsfeindlicher Vorfälle*^31^, from which the ARVIG dataset^6^ is webscraped and on which anti-refugee-violence research such as Dancygier *et al*.^32^ draws; the Amadeu Antonio Stiftung’s record of deaths caused by right-wing violence^33^; and the counseling-centre records used here. This dataset improves on these sources by coding violence across the full range of far-right motives rather than a single target group, by integrating violence with music and protest in one county-year panel, and by harmonising all records to fixed 2024 county boundaries. The alternatives retain distinct strengths and are best read as complements rather than substitutes: ACLED^5^ offers cross-national comparability, near-real-time coverage, and actor-coded events from which organisers can sometimes be identified. However, it includes data on Germany only from 2020, is drawn exclusively from news media, and carries no far-right-specific motive code. The *Chronik*/ARVIG line provides a longer anti-refugee benchmark, but is limited to a single motive category. The Amadeu Antonio list provides the longest series for cases of far-right violence, but is limited to homicide. Two cross-national collections set our German focus in a wider European frame: the C-REX *Right-Wing Terrorism and Violence* (RTV) dataset^11^ offers the most systematic record of severe and lethal far-right violence across nineteen West European countries since 1990, but by design captures only the most severe incidents and neither protest nor music. The *Comparative Far-Right Protest* line^34,35^ codes far-right protest events across European countries from newspaper sources, complementing our protest stream transnationally while sharing the media-selection limits of press-based event data. FARAC is therefore best understood not as a substitute for ACLED or these civil-society and cross-national collections, but as a complementary, differently structured measurement tool: a far-right-specific, county-level integration of three activism types for Germany.

**Table 3:** FARAC in relation to existing data sources on far-right activism and violence.

Source	Unit	Coverage	Captures	Collection & scope	Main trade-off vs FARAC
FARAC	events + county-year panel	Germany; panel 2013–2024 (400 counties, 2024 boundaries); event series from 2002 (violence), 2005 (protest), 2009 (music)	music, protest, violence (all far-right motives)	parliamentary inquiries + counseling centres + Kanol *et al*.; far-right-specific	—
Chronik flüchtlingsfeindlicher Vorfälle	incident	Germany, 2015–2023	anti-refugee incidents (attacks, demonstrations, agitation)	media reports (Amadeu Antonio Stiftung + PRO ASYL); anti-refugee only	broader incident threshold, but single motive (anti-refugee)
ARVIG (Benček & Strasheim^6^	event (geocoded)	Germany, 2014–2017	anti-refugee violence and demonstrations	webscrape of the Chronik; anti-refugee only	clean geocoded benchmark, but single motive, short window, a subset of the Chronik
Amadeu Antonio Stiftung – Todesopfer rechter Gewalt	fatal case	Germany, since 1990	lethal far-right violence only	media + counseling centres + journalists; victim-centred	longest series, but homicides only
ACLED	event (geolocated)	global; Germany from 2020	protests, riots, political violence (generic)	news media; actor-coded; no far-right motive code	cross-national and near-real-time, but media-selection bias, Germany only from 2020, far-right events not separable by code
RTV (C-REX; Ravndal *et al*.)	incident (geolocated)	19 West European countries, since 1990	severe and lethal far-right violence, attack plots, arms caches (all target groups)	open sources (news, court, NGO) + national experts; severe-violence threshold	cross-national and the longest severe/lethal series, but severe violence only, no protest or music, not a county panel
FARPE / CFP (Castelli Gattinara *et al*.)	protest event	11 European countries, 2008–2018 (FARPE); extended to 2008–2021 (CFP)	far-right protest only	newspaper protest-event analysis; one quality paper per country	cross-national far-right protest, but protest only, press-sourced (media-selection bias), CFP not yet public

Coverage gives each source's full span; for FARAC's coverage of each activism type over time, see Fig. 5.

Sources: Chronik flüchtlingsfeindlicher Vorfälle (Mut gegen rechte Gewalt, via Internet Archive); ARVIG (Benček & Strasheim^6^); Amadeu Antonio Stiftung, Todesopfer rechter Gewalt; ACLED (Raleigh *et al*.); RTV (Ravndal *et al*.^11^, C-REX); FARPE (Castelli Gattinara, Froio & Pirro^34^) and CFP (Castelli Gattinara & Segers^35^).

## Data Availability

The dataset generated in this work is deposited on Zenodo under a CC-BY 4.0 licence, with the registered DOI https://doi.org/10.5281/zenodo.21041488^25^. The deposit contains the harmonized county-level panel (far_right_activism_panel.csv); event-level records of far-right music events (music_events.csv) and protests (protest_events.csv); the monthly county-level music-and-protest file (music_protest_monthly.csv); county-year aggregated violence data from counseling centres (violence_county_year.csv); state-year official government violence statistics (official_violence.csv); and a GeoPackage with 2024 county boundaries for spatial analysis (county_boundaries_2024.gpkg). Codebooks describing all variables are provided alongside the data files. Details on the input data and their sources are given in the Methods (see “Data sources and provenance”).

## Code availability

We processed and analyzed all data through a transparent R-based workflow^36^ and maintained the results in publicly accessible repositories. To digitize, clean, and supplement the raw government responses, we built an R targets pipeline^37^ using the tabulapdf^26^, tidyverse^38^, and sf^39^ packages. The complete replication code is available at: https://github.com/markushlang/farac/. The raw government PDF responses on which the pipeline operates are publicly available from the Bundestag Documentation and Information System (DIP; https://dip.bundestag.de).
